# Covalent Bonding of MXene/COF Heterojunction for Ultralong Cycling Li-Ion Battery Electrodes

**DOI:** 10.3390/molecules29122899

**Published:** 2024-06-18

**Authors:** Yongbiao Liu, Yang Song, Quanbing Lu, Linsen Zhang, Lulu Du, Shiying Yu, Yongshang Zhang

**Affiliations:** 1 Shanghai Putailai New Energy Technology Co., Ltd., Shanghai 210315, China; 2 Henan Electric Power Transmission & Transformation Construction Co., Ltd., Zhengzhou 450001, China; 3 College of New Energy, Zhengzhou University of Light Industry, Zhengzhou 450001, China; 4 Henan International Joint Laboratory of Ceramic Energy Materials, Zhengzhou 450001, China

**Keywords:** 3D COFs, Ti_3_C_2_ MXenes, Li-ion battery, DFT calculations

## Abstract

Covalent organic frameworks (COFs) have emerged as promising renewable electrode materials for LIBs and gained significant attention, but their capacity has been limited by the densely packed 2D layer structures, low active site availability, and poor electronic conductivity. Combining COFs with high-conductivity MXenes is an effective strategy to enhance their electrochemical performance. Nevertheless, simply gluing them without conformal growth and covalent linkage restricts the number of redox-active sites and the structural stability of the composite. Therefore, in this study, a covalently assembled 3D COF on Ti_3_C_2_ MXenes (Ti_3_C_2_@COF) is synthesized and serves as an ultralong cycling electrode material for LIBs. Due to the covalent bonding between the COF and Ti_3_C_2_, the Ti_3_C_2_@COF composite exhibits excellent stability, good conductivity, and a unique 3D cavity structure that enables stable Li^+^ storage and rapid ion transport. As a result, the Ti_3_C_2_-supported 3D COF nanosheets deliver a high specific capacity of 490 mAh g^−1^ at 0.1 A g^−1^, along with an ultralong cyclability of 10,000 cycles at 1 A g^−1^. This work may inspire a wide range of 3D COF designs for high-performance electrode materials.

## 1. Introduction

In the past few decades, due to the excessive consumption of fossil fuels and the increasing frequency of human activities, the global environment has deteriorated [1,2,3]. In response to this, global goals for peak carbon emissions and carbon neutrality have been established, and there is a strong emphasis on promoting the use of green energy such as wind, tidal, and solar power [4,5,6]. However, due to the intermittent nature of renewable energy, efficient energy storage systems are needed to store and utilize it. Among the various energy storage systems, LIBs have dominated the market as a power supply system for smart devices, electric vehicles (EVs), and more [7,8,9]. Nevertheless, with the rapid evolution of EVs and smart grids, there are higher demands on the performance indicators of LIBs, particularly in terms of energy density and power density [10,11,12]. Therefore, there is an urgent need to explore new electrodes with high capacity to meet these increasing requirements [13,14]. Currently, the most frequently used anode material for LIBs is graphite, but it has nearly achieved its theoretical specific capacity of 372 mAh g^−1^ [14,15]. The limited specific capacity of the anode material poses a constraint on LIBs to achieve an energy density of 300 Wh kg^−1^ [16,17]. This inability to meet the demands of the new energy market highlights the importance of designing and developing new anode materials that offer both high specific capacity and high-rate performance [18,19,20].

COFs are a new type of polymer constructed using modular organic building blocks linked by strong covalent bonds and have gained attention for their various applications in areas such as gas storage, semiconductors, catalysis, and drug delivery [21,22,23,24]. Recently, COFs have shown excellent potential as electrode materials due to their excellent structure and thermal stability. For example, Huang et al. [25] have successfully synthesized a novel Janus dione-based COF with well-organized 2D crystalline structures and abundant redox-active sites which makes it highly suitable as an electrode material for LIBs. The COF-based cathode demonstrated a significant capacity of 338 mAh g^−1^ at 0.1 C. Another study by Lee et al. [26] involved the fabrication of redox-active COF materials capable of accommodating up to 30 Li^+^ ions, resulting in a remarkable specific capacity of 764.1 mAh g^−1^. Both these studies exemplify the potential of COFs as active materials for battery electrodes. However, it is worth noting that despite these advancements, there are still challenges to be addressed. The tightly packed two-dimensional layer structures of COFs often lead to the burial of interior accessible active sites, limiting their full utilization and resulting in reduced reversible capacity [27,28]. Additionally, COFs face limitations in terms of electronic conductivity and sluggish lithium diffusion kinetics, which will hinder their broader applications in energy storage [29].

Ti_3_C_2_ MXene is a two-dimensional layered transition metal carbide material with a graphite-like structure [30,31,32]. It is primarily produced through etching of the precursor material Ti_3_AlC_2_ [33,34,35]. The unique layered structure of Ti_3_C_2_ gives it exceptional electrical conductivity, high surface area, and chemical stability, thanks to its specific elemental composition and surface functional groups [36,37]. As a result, Ti_3_C_2_ has found extensive applications as an active material in energy storage systems. Considering the outstanding properties of Ti_3_C_2_ and the limitations of COFs, combining these two materials appears to be a promising approach for fabricating high-performance electrode materials [38,39]. By leveraging the conductivity and large surface area of Ti_3_C_2_ along with the unique features of COFs, it is possible to develop electrode materials with enhanced performance for various energy storage applications. However, simply gluing them without conformal growth and covalent linkage restricts the number of redox-active sites and the structural stability of the composite [40,41,42,43,44,45].

In this study, a covalently assembled 3D COF on Ti_3_C_2_ MXene (Ti_3_C_2_@COF) is synthesized and serves as an ultralong cycling electrode material for LIBs. To achieve this, the COF and Ti_3_C_2_ are connected through covalent linking between aldehydes and amino groups, resulting in the formation of a Ti_3_C_2_@COF composite. Due to the covalent bonding between the COF and Ti_3_C_2_, the Ti_3_C_2_@COF composite exhibits excellent stability, good conductivity, and a unique 3D cavity structure that enables stable Li^+^ storage and rapid ion transport. The 3D COFs offer several advantages over their 2D counterparts, including higher specific surface areas and more pore structures. The unique structure of Ti_3_C_2_ in the composite plays a key role in shortening the diffusion pathway for ions and electrons and providing a larger surface area for the combination with the 3D COF. Through DFT calculations, it is confirmed that the 3D COF can accommodate four Li^+^ ions within its 3D pore channels and store an additional three Li^+^ ions at the interface between the COF and Ti_3_C_2_. This exceptional Li^+^ storage capability of the Ti_3_C_2_@COF anode is further demonstrated experimentally. The Ti_3_C_2_@COF anode exhibited a high capacity of 490 mAh g^−1^ at a rate of 0.1 A g^−1^ and demonstrated an extraordinarily long cycling life, maintaining a capacity of 180 mAh g^−1^ over 10,000 cycles at a rate of 1 A g^−1^. This work expands the diversity of 3D COFs in the field of energy storage, showcasing the potential of 3D COFs as a high-performance electrode material for LIBs.

## 2. Results and Discussion

**Figure 1:**
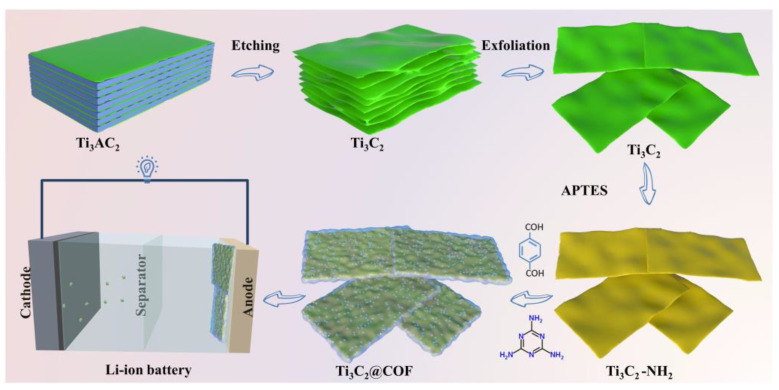
Schematic illustration of the synthesis of Ti_3_C_2_@COF composite.

The fabrication of Ti_3_C_2_@COF composite is schematically shown in Figure 1. Briefly, Ti_3_C_2_T*_x_* nanosheets were exfoliated from the MAX phase and aminated using 3−aminopropyl triethoxysilane (APTES). Then, the 3D COF layer grew on the amino-grafted Ti_3_C_2_ (Ti_3_C_2_–NH_2_) surface via the covalent linking between the aldehydes and amino groups of the respective monomers to obtain a Ti_3_C_2_@COF nanoarchitecture. Specifically, pre-grafting homologous APTES onto the surface of Ti_3_C_2_ nanosheets is crucial for controlling the growth of uniform crystal 3D COF materials. Due to the large number of terminal groups on the surface of Ti_3_C_2_, such as –F, –O, and –OH, hydrogen bonding interactions ensure that the APTES molecules tightly bind to Ti_3_C_2_, resulting in modified Ti_3_C_2_ nanosheets with rich –NH_2_ groups. The introduction of Ti_3_C_2_ is aimed at enhancing the electronic conductivity of the obtained Ti_3_C_2_@COF composite. Notably, the unique 2D lamellar structure of Ti_3_C_2_ can shorten the diffusion pathway of ions/electrons and offer more surface area to combine the 3D COF with high Li storage capacity.

**Figure 2:**
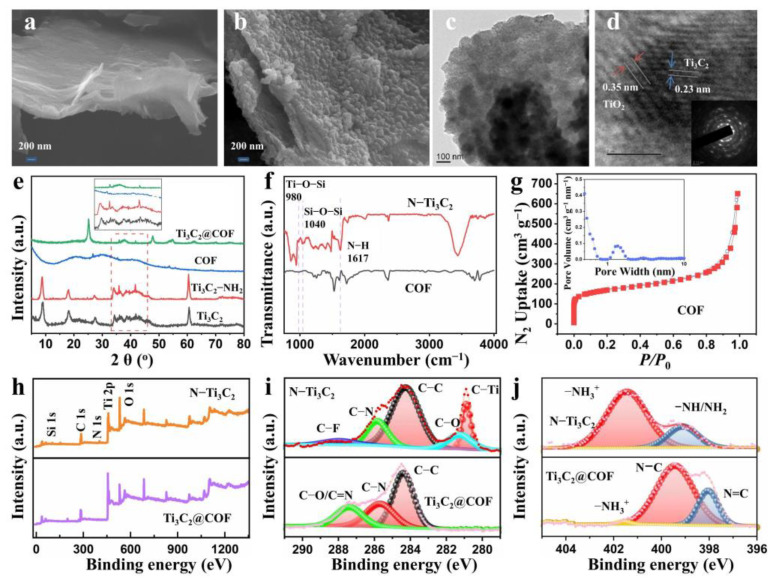
(**a**,**b**) SEM images of Ti_3_C_2_ and Ti_3_C_2_@COF composite. (**c**,**d**) TEM images of Ti_3_C_2_@COF composite. (**e**) XRD patterns of Ti_3_C_2_, Ti_3_C_2_–NH_2_, COF, and Ti_3_C_2_@COF. (**f**) FTIR spectra of COF and Ti_3_C_2_–NH_2_ nanosheets. (**g**) Nitrogen sorption isotherms of COF materials. (**h**) XPS wide spectra of Ti_3_C_2_–NH_2_ and Ti_3_C_2_. (**i**,**j**) XPS C1s and N1s core level spectra resolving results of Ti_3_C_2_–NH_2_ and Ti_3_C_2_@COF.

To confirm the morphological changes during the synthesis process, SEM and TEM were conducted on the Ti_3_C_2_ and Ti_3_C_2_@COF composite, as shown in Figure 2a–d, Figures S2 and S3. Figure 2a shows the morphology of Ti_3_C_2_ nanosheets, which reveal a 2D lamellar structure with a large surface area. Figure S2 shows the aminated lamellar Ti_3_C_2_ without any particles on the surface. In addition, the EDS elemental mapping of amino-grafted Ti_3_C_2_ nanosheets confirms the uniform distribution of C, N, Ti, and O elements, which demonstrates the successful amino groups grafted on the Ti_3_C_2_ surface (Figure S3). SEM image of Ti_3_C_2_@COF composite demonstrated the homogeneous and rough surface of COFs with granular morphology (Figure 2b). The assembly of 3D COFs on the Ti_3_C_2_ surface could be seen in TEM images (Figure 2c,d). Notably, the Ti_3_C_2_@COF composite still retains a 2D lamellar structure. This phenomenon demonstrates the grafted amino can firmly anchor 3D COF on the Ti_3_C_2_ surface. A high-resolution TEM image of the COF@Ti_3_C_2_ composite is shown in Figure 2d. The lattice spacing values of 0.23 and 0.35 nm correspond to the (104) surface of Ti_3_C_2_ and the (101) surface of TiO_2_, respectively. Furthermore, the (104) surface of TiO_2_ observed in TEM is attributed to the slight oxidation of the Ti_3_C_2_ material due to the high-temperature effect.

Figure 2e shows the XRD patterns of Ti_3_C_2_, Ti_3_C_2_–NH_2_, COF, and Ti_3_C_2_@COF composite. The characteristic peak of 9.0° corresponds to the (002) plane of Ti_3_C_2_. Importantly, the (002) plane corresponding to Ti_3_C_2_ in the Ti_3_C_2_–NH_2_ composite can be detected in the XRD pattern, which proves that the amino-functionalization process does not affect the structure of Ti_3_C_2_ nanosheets. After the hydrothermal reaction, new peaks appeared at 25.3°, 37.9°, 47.8°, 53.8°, 54.8°, and 62.5° corresponding to the (101), (004), (200), (105), (211), and (304) planes of anatase TiO_2_, respectively. Moreover, the peaks at 36° and 42° corresponding to Ti_3_C_2_ in the magnified XRD spectra remained, confirming the formation of the COF@Ti_3_C_2_ hybrid structure. The surface area and pore volume play a critical role in the electrochemical performance of an electrode. Therefore, the COF and Ti_3_C_2_’s specific surface area and pore properties were analyzed using N_2_ adsorption-desorption plots, and the obtained data are presented in Figure 2g and Figure S4. The 3D COF shows a high surface area of 546.0 m^2^ g^−1^ and pore volume of 1.0 cm^3^ g^−1^, but the Ti_3_C_2_ shows a low surface area of 6.0 m^2^ g^−1^ and pore volume of 0.03 cm^3^ g^−1^.

To demonstrate the successful functionalization of MXenes and the successful composite of 3D COF materials, the surface group evolution was examined through ATRFTIR and XPS. The covalent amination of MXene was confirmed by the presence of the NH_2_ band in FTIR. As depicted in Figure 2f, the appearance of peaks at 1040 and 980 cm^−1^ corresponding to the Si–O–Si and Ti–O–Si bonds in the Ti_3_C_2_ nanosheets confirms the covalent reaction between APTES and MXene. Additionally, the peak at 1617 cm^−1^, which is attributed to the bending mode of the N–H bond of the primary amine units, confirms the successful amino-functionalization of the MXene nanosheet. The infrared spectra of 3D COF and Ti_3_C_2_@COF are shown in Figure S5. A peak at 1550 cm^−1^ in the FTIR spectra is associated with the triazine ring structure within the COF material. As shown in Figure S5, the FTIR spectra of the COF and Ti_3_C_2_@COF composite exhibit a peak at 1550 cm^−1^, indicating the successful synthesis of the COF and Ti_3_C_2_@COF composite. Figure 2h shows the XPS wide spectra of Ti_3_C_2_–NH_2_ and Ti_3_C_2_, which confirm the appearance of N and Si elements in the Ti_3_C_2_–NH_2_. This result is consistent with FTIR spectra, confirming the success of the amination process. The C1s core level spectra resolving results reveal the C–Ti bond from Ti_3_C_2_ nanosheets and the C–F and C–O bonds which derive from the etching process. The –NH_3_, –NH/NH_2_, and N–C bonds in N 1s and C1s correspond to the amino group grafted on the COF surface. The N=C bond in N 1s and C 1s proves the 3D COF and Ti_3_C_2_ incorporated successfully. The above evidence indicates the successful production of Ti_3_C_2_@COF composite.

**Figure 3:**
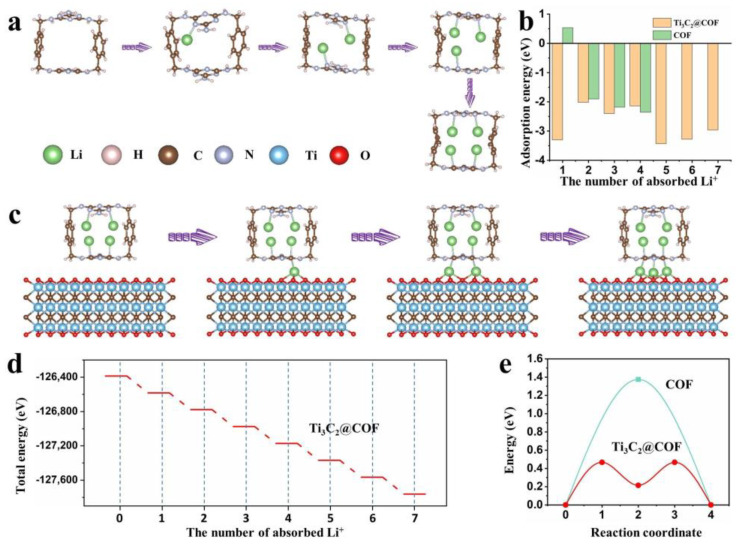
Structural evolution of (**a**) COF and (**c**) Ti_3_C_2_@COF electrodes during the lithiation procedures. (**b**) The Li adsorption energies in the lithiation process for the COF and Ti_3_C_2_@COF. (**d**) Proposed lithiation pathway for the Ti_3_C_2_@COF electrode. The right axis shows the redox potential versus Li^+^/Li, and the left axis shows the total energy of various lithiated Ti_3_C_2_@COF structures. (**e**) Energy profiles of the barrier for Li^+^ ion diffusion on the interface of COF and Ti_3_C_2_ and the surface of COF.

To better understand the redox mechanism of the Ti_3_C_2_@COF electrode, lithiated models of COF with four Li^+^ ions and Ti_3_C_2_@COF with seven Li^+^ ions were first simulated, and the adsorption energy, free energy, and total energy of Li^+^ ions in these models were calculated by DFT calculation, as shown in Figure 3. According to the calculated results, Li^+^ ions most prefer bonds with N or C atoms and insert into the 3D cavity structure of COF material, forming COF + 4Li. The structural evolution of COF with different numbers of Li^+^ ions during the lithiation procedure is shown in Figure 3a. The Ti_3_C_2_@COF composite could receive another 3Li^+^ ions at the interface of COF and Ti_3_C_2_ materials, and the structural evolution of Ti_3_C_2_@COF composite during the lithiation procedure is shown in Figure 3c. These results prove that the combination of 3D COF and Ti_3_C_2_ materials could offer more active sites to store Li^+^ ions. As shown in Figure 3b, the Ti_3_C_2_@COF shows smaller adsorption energy than that of pure COF. This reveals that the combination of COF and Ti_3_C_2_ could speed up Li adsorption in the COF, which will contribute to better rate performance. The free energy of the COF and Ti_3_C_2_@COF electrode with different insert numbers of Li^+^ ions was calculated and shown in Figure S6. During the discharge process, the Gibbs free energy of the COF electrode increases during the initial insertion of the first Li^+^, but subsequently decreases as more lithium ions are inserted. This indicates that the adsorption of Li^+^ ions on the active site of the COF is initially unstable, but becomes more stable with an increasing number of lithium ion insertions (Figure S6). It is surprising that the Gibbs free energy of the Ti_3_C_2_@COF electrode continuously decreases as Li^+^ ions are inserted during the discharge process. This indicates that Li^+^ ions can stably adsorb onto the composite material. This result demonstrates that the introduction of Ti_3_C_2_ enhances the stability of COF in Li^+^ storage (Figure S6c). The total energy for Ti_3_C_2_@COF is −1.263 × 10^5^ eV. The energies of the lithiation process for the 3D COF cavity and the heterojunction of COF and Ti_3_C_2_ decreased to −1.269 × 10^5^ and −1.277 × 10^5^ eV after inserting four and three Li^+^ ions (Figure 3d). The total energy shows a downward trend in the lithiation process for the Ti_3_C_2_@COF configuration indicating that Li adsorption on all these steps is thermodynamically favorable. Furthermore, the energy barrier for Li^+^ ion diffusion was calculated, as shown in Figure 3e. The calculated results confirm the low Li^+^ ions diffusion energy barrier of 0.46 eV on the interface of COF and Ti_3_C_2_, compared with a fresh COF electrode (1.37 eV). In summary, DFT calculation confirms that the Ti_3_C_2_ can enhance the lithium storage stability of COF electrodes, increase the lithium storage capacity of composite electrodes, and promote the rapid transfer of Li^+^ ions. Notably, the 3D COF layer grew on the amino-grafted Ti_3_C_2_ (Ti_3_C_2_–NH_2_) surface via the covalent linking between the aldehydes and amino groups of the respective monomers to obtain a Ti_3_C_2_@COF nanoarchitecture. Therefore, the COF and Ti_3_C_2_ are connected by an amino group, which means there is a spacing for the amino group between COF and Ti_3_C_2_ composite. In the process of building the model, to simplify the configuration and accelerate the analysis process, we omitted the amino group and retained a gap between COF and Ti_3_C_2_, which is about 7 Å. In actual situations, there are some amino groups in the spacing between COF and Ti_3_C_2_, which would act as a barrier toward Li diffusion and affect the corresponding adsorption energies to some extent. However, the simplified configuration of COF and Ti_3_C_2_ shows a clear trend of promoting Li^+^ transport and enhancing lithium storage capacity, which is consistent with the electrochemical performance testing results of the Ti_3_C_2_@COF nanoarchitecture. Therefore, simplified configurations can to some extent reflect the actual situations of materials.

**Figure 4:**
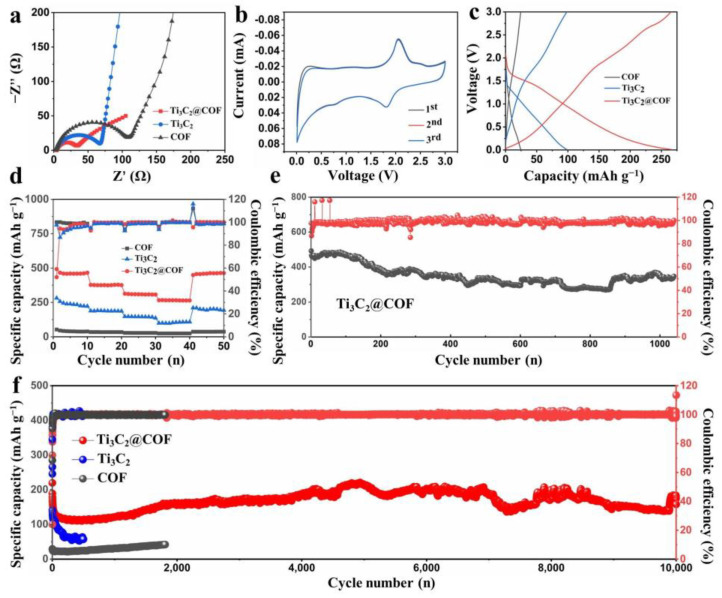
(**a**) EIS curves of COF, Ti_3_C_2_@COF, and Ti_3_C_2_ electrodes. (**b**) CV curves of Ti_3_C_2_@COF electrode. (**c**) Galvanostatic charge-discharge curves and (**d**) rate performance of the COF, Ti_3_C_2_@COF, and Ti_3_C_2_ electrodes. (**e**) Long-term cycling stability of Ti_3_C_2_@COF electrode at 0.1 C. (**f**) Stability test of COF, Ti_3_C_2_@COF, and Ti_3_C_2_ electrodes at 1 A g^−1^.

To illustrate the electrochemical performances of Ti_3_C_2_@COF composite, the EIS, CV, rate, and long-term cycle performance were investigated, as shown in Figure 4. The EIS results show that the Ti_3_C_2_@COF composite has lower charge transfer resistance than the pure Ti_3_C_2_ and COF electrodes, which is attributed to the combination of better electro-conductivity of Ti_3_C_2_ nanosheets and the unique 3D pore structure of COF (Figure 4a). Figure 4b and Figure S7 show the CV test of the Ti_3_C_2_@COF and COF, Ti_3_C_2_ electrode at 0.1 mV s^−1^. The Ti_3_C_2_@COF electrode shows distinct redox peaks at 1.80 and 2.1 V which are assigned to the reversible lithiation/delithiation reactions. In particular, a prominent peak at 1.8 V during the discharge process of Ti_3_C_2_@COF is attributed to the lithium reaction with the C=N/C–N groups, while the peak at 0.7 V assigned to the insertion of Li^+^ ions into the heterojunction of Ti_3_C_2_ and COF materials. Figure S7a shows the CV curves of pure COF anode, the two peaks at 1.5 and 0.6 V ascribed to the reduction reaction of C=N and C–N with Li^+^. The CV curves of Ti_3_C_2_ display two peaks in the first cycle, which is ascribed to an irreversible reaction of the formation of a solid electrolyte interface (SEI) film on the electrode surface (Figure S7b). Compared with the Ti_3_C_2_@COF electrode, the COF electrode shows a weak CV-responsive signal and the Ti_3_C_2_ anode has too much irreversible capacity.

The specific capacities of prepared electrodes were tested using galvanostatic charge-discharge curves at 1 A g^−1^, as demonstrated in Figure 4c. The 3D COF electrode only delivers a low capacity of 24 mAh g^−1^, which is attributed to the low electrical conductivity and dense structure of the COF itself. The specific capacity of the Ti_3_C_2_ electrode is only 98 mAh g^−1^. Such a low specific capacity can be attributed to the low-capacity nature of Ti_3_C_2_. Notably, the Ti_3_C_2_@COF anode delivers a high capacity of 266 mAh g^−1^ at the same current density, indicating that more redox active sites of Ti_3_C_2_@COF are utilized due to the enhanced conductivity and more exposed 3D cavity structure. Then, the rate capability performance of COF, Ti_3_C_2_, and the Ti_3_C_2_@COF electrodes were investigated over the range from 0.1 to 1 A g^−1^ (Figure 4d). For the Ti_3_C_2_@COF, average capacities of 490, 380, 312, and 266 mAh g^−1^ can be obtained under 0.1, 0.2, 0.5, and 1 A g^−1^, respectively. In addition, the capacity recovers to nearly 465 mAh g^−1^ after the current density returns to 0.1 A g^−1^, which suggests good electrochemical reversibility of Ti_3_C_2_@COF anode. However, the COF and Ti_3_C_2_ electrodes show low capacity and inferior rate performance. Further evidence for the electrochemical stability of Ti_3_C_2_@COF anodes is provided by the similarity in the shape of the charge/discharge curves. This indicates the absence of significant polarization and indirectly demonstrates the excellent transport properties of both Li ions and electrons within the Ti_3_C_2_@COF anodes, as shown in Figure S8. The long-term cycling performance of Ti_3_C_2_@COF anodes was further evaluated at 0.1 C, as shown in Figure 4d. It is worth noting that Ti_3_C_2_@COF anodes still have a high capacity of 355 mAh g^−1^ even after 1000 cycles. Nevertheless, as can be seen in Figure 4e, the excellent cycling stability of Ti_3_C_2_@COF anodes is also verified by electrochemical measurements at high rates of 1 A g^−1^, showing a high capacity of 180 mAh g^−1^ even after 10,000 cycles, representing one of the best COF-based electrodes in LIBs in terms of both long cycling stability and high capacity.

**Figure 5:**
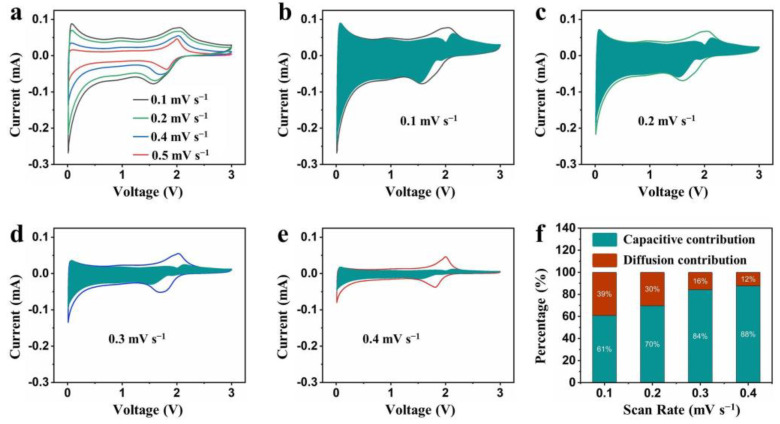
(**a**) CV curves of Ti_3_C_2_@COF anode at different scan rates. (**b**–**f**) The calculated proportion of diffusion and capacitive contribution of Ti_3_C_2_@COF anode at different scan rates.

The CV test at 0.1–0.5 mV s^−1^ was conducted to investigate the kinetic behavior of Li^+^ storage in the Ti_3_C_2_@COF anodes, as shown in Figure 5. Equations (1) and (2) were used to express the relationship between the peak current and the sweep rate. Here, *i* represents the peak current, *v* stands for the sweep rate, and *a* and *b* denote adjustable parameters.

*I* = *av*^*b*^ (1)

log(*i*) = *b* log(*v*) + log(*a*) (2)

*i* = *k*_1_*v* + *k*_2_*v*^1/2^ (3)

We further utilize Cook’s analytical method to ascertain the collective impact of pseudo-capacitors across different sweep rates. The total current at a constant potential is segmented into a pseudocapacitive mechanism (*k*_1_*v*) and an ion diffusion process (*k*_2_*v*^1/2^). The calculation of the pseudocapacitive contribution is determined by the following Equation (4).

*i* = *k*_1_*v* + *k*_2_*v*^1/2^ (4)

As illustrated in Figure 5b–f, the proportion of the capacitive contribution increases as the scan rate increases. This suggests that the insertion/extraction of Li^+^ in the Ti_3_C_2_@COF electrode relied on a rapidly kinetic pseudocapacitive process, which improved the rate performance of the Ti_3_C_2_@COF electrode.

## 3. Materials and Methods

### 3.1. Preparation of Ti_3_C_2_ MXenes 

To synthesize Ti_3_C_2_T_x_ MXene, the selective etching of aluminum from Ti_3_AlC_2_ is carried out. Firstly, 1 g of LiF is dissolved in 9 mol L^−1^ hydrochloric acid. Then, 1 g of Ti_3_AlC_2_ is gradually added to the solution, and the mixture is stirred at 35 °C for 24 h. Afterward, the acidic suspension is washed with deionized water through centrifugation at 3500 rpm until the pH of the solution exceeds 6. Following the washing step, the suspension is subjected to ultrasonic dispersion for 2 h, ensuring a uniform dispersion. The mixture is then shaken and subjected to centrifugation, and the supernatant is collected for further use. 

### 3.2. Synthesis of COF and Ti_3_C_2_@COF Composite

The next step involves adding 3−aminopropyl triethoxysilane (APTES) to the obtained suspension and stirring it for 12 h. The resulting precipitate is then washed several times with ethanol and ultrapure water. Subsequently, it undergoes freeze-drying to obtain amino−grafted Ti_3_C_2_. The functionalized Ti_3_C_2_ is then combined with a solution consisting of melamine (3.5 mg) and terephthalaldehyde (5.5 mg) dissolved in dimethyl sulfoxide (3 mL). This mixture is placed in a 20 mL autoclave and heated to 180 °C for 10 h. After cooling down to room temperature, the Ti_3_C_2_@COF composite is filtered and washed using acetone, tetrahydrofuran, and dichloromethane. The resulting powder, which appears white, is then dried overnight at a temperature of 45 °C in a vacuum oven. Furthermore, pure COF (Schiff base networks (SNW)) was synthesized using the same procedure but without Ti_3_C_2_ supernatant. Briefly, melamine (3.5 mg) and terephthalaldehyde (5.5 mg) were dissolved in dimethyl sulfoxide (3 mL) and placed in a 20 mL autoclave. Then, the mixture was heated to 180 °C for 10 h. The synthesis route and chemical structure of COF are shown in Figure S1 in the Supporting Information.

### 3.3. Materials Characterization

The surface morphology of the prepared samples was analyzed using scanning electron microscopy (SEM) equipped with energy-dispersive X-ray spectroscopy (EDS) analysis. For this analysis, a FE-SEM (JSM-7001F, JEOL Ltd., Tokyo, Japan) was utilized. Additionally, the samples were examined using transmission electron microscopy (TEM) analysis with a JEM-2100 TEM instrument (JEOL Ltd., Tokyo, Japan). To investigate the structural properties of the samples, X-ray diffraction (XRD) analysis was performed using a D8-ADVANCE instrument (Bruker, Billerica, MA, USA) with Cu Kα radiation. Fourier-transform infrared spectroscopy (FT-IR) spectra were recorded using a JASCO model FT IR-6100 infrared spectrometer (JASCO, Oklahoma City, OK, USA). For further characterization of the samples, X-ray photoelectron spectroscopy (XPS) analysis was conducted using a Thermo Scientific K-Alpha energy spectrometer (Thermo Fisher Scientific, Waltham, MA, USA). The nitrogen adsorption and desorption isotherms were analyzed using an ASAP 2460 instrument (Micromeritics Instrument Corporation, Norcross, GA, USA) at a temperature of 77 K.

### 3.4. Electrochemical Characterization

The electrochemical properties were conducted by a CR2016 coin-type cell in a glove box. The working electrodes were prepared by mixing 80 wt% active materials, 10 wt% Super P, and 10 wt% poly(vinylidene fluoride) (PVDF). Coin-type test cells were assembled in an argon-filled glove box with Li foil as the counter electrode, Celgard 2400 as the separator, and 1 M LiPF_6_ in ethylene carbonate/dimethyl carbonate (EC/DMC vol 1:1) as the electrolyte. Cyclic voltammetry (CV) (0.01–3 V, 0.1 mV s^−1^) and electrochemical impedance spectroscopy (EIS) (0.01–100,000 Hz, 5 mV) were conducted using a CHI660E electrochemical workstation. 

### 3.5. DFT Calculations

All DFT calculations employing spin polarization were carried out using the Vienna Ab Initio Simulation Package (VASP 6.1.0) [46]. The Perdew–Burke–Ernzerhof (PBE) functional under the generalized gradient approximation (GGA) was employed to represent electron exchange-correlation, and the projector augmented wave (PAW) method was used to define the pseudo-potentials [47]. The structure model of COF is built from crystalline structures in the Inorganic Crystal Structure Database (ICSD) [48]. A 5 × 5 × 1 supercell of the Ti_3_C_2_O_2_ (001) facet was utilized. The Brillouin zones were sampled using the Monkhorst–Pack scheme with a 3 × 3 × 1 k-point grid. The DFT-D3 correction with the Grimme scheme was applied to consider the dispersion interaction [49]. The energy profiles of the barrier for Li^+^ ion diffusion on the interface of COF and Ti_3_C_2_ and the surface of COF were simulated by the climbing-image nudged elastic band (CI-NEB) method implemented in the VASP [50]. In this work, the Ti_3_C_2_ is obtained by selectively etching Al element from the precursor Ti_3_AlC_2_ in a hydrofluoric acid-containing solution. This method inevitably introduces various content-uncontrollable terminations on the surface of Ti_3_C_2_, such as –O, –OH, and –F. Therefore, choosing Ti_3_C_2_O_2_ as the structure model to simulate the lithiation procedures and Li^+^ diffusion in the Ti_3_C_2_@COF electrode is closer to the real environment, a more realistic reflection of the transport and embedding process of Li^+^ in electrodes.

The Li adsorption energy (*E*_ads_) is calculated by the following Equation (5):

*E*_ads_= (*E*_ta_ − *E*_tb_ − *nE*_isolated Li_)/*n* (5)

where *E*_tb_ and *E*_ta_ are the total energies of COF (or Ti_3_C_2_@COF) and a Li-adsorbed COF (or Ti_3_C_2_@COF), respectively. *E*_isolated Li_ is the energies of an isolated Li atom and *n* is the number of adsorbed Li atoms.

## 4. Conclusions

In conclusion, a Ti_3_C_2_@COF electrode for LIBs was prepared by covalent bonding which possesses high surface areas and abundant pore channels, making it well-suited for storing and transferring Li ions. The unique structural advantages of 3D COFs, combined with the robust skeleton of Ti_3_C_2_, allow a Ti_3_C_2_@COF electrode to obtain high capacities, excellent rate performance, high utilization of the active site, and good cyclability. This represents one of the best overall performances among the COF-based electrodes reported thus far. The pathway for the storage of Li^+^ in the Ti_3_C_2_@COF electrode has been elucidated through a series of electrochemical analyses and DFT calculations. The Ti_3_C_2_-supported 3D COF nanosheets deliver a high specific capacity of 490 mAh g^−1^ at 0.1 A g^−1^ between 0.01–3 V, which could offer a much higher energy density than the commercial graphite anode. However, the preparation procedure of the Ti_3_C_2_@COF anode needs to be further improved and simplified, due to the complex preparation process of Ti_3_C_2_ nanosheets will increase the cost of large-scale production. This work would inspire a wide range of 3D COF designs for high-performance electrode materials. These results not only present a new 3D COF anode with better electrochemical performances but also provide a platform for designing and manufacturing high-performance electrode materials for energy storage.

## Data Availability

The data presented in this study are available on request from the corresponding author.

## Supplementary Materials

The following supporting information can be downloaded at: https://www.mdpi.com/article/10.3390/molecules29122899/s1: Figure S1: The synthesis route and chemical structure of COF; Figure S2: SEM images of Ti_3_C_2_–NH_2_; Figure S3: Elemental mapping of Ti_3_C_2_–NH_2_; Figure S4: Nitrogen sorption isotherms of Ti_3_C_2_ materials; Figure S5: FTIR spectra of COF and Ti_3_C_2_@COF composite; Figure S6: (a) Proposed lithiation pathway for the COF electrode. (b) The Gibbs free energies for lithiated models of COF + xLi (x=1-4). (c) The Gibbs free energies for lithiated models of Ti_3_C_2_@COF + xLi (x=1-7); Figure S7: CV curves of (a) COF and (b) Ti_3_C_2_ electrode; Figure S8: Galvanostatic charge-discharge curves of Ti_3_C_2_@COF electrode at different current density.
