# Supplementary material for: Covalent Bonding of MXene/COF Heterojunction for Ultralong Cycling Li-Ion Battery Electrodes
Source: Molecules. 2024 Jun 18;29(12):2899. doi: 10.3390/molecules29122899 (PMC11207039; doi:10.3390/molecules29122899)
Supplement: Supplementary file 1 [file molecules-29-02899-s001.zip › molecules-3007248-supplementary.pdf]

molecules

Supporting Information

Covalent Bonding of MXene/COF Heterojunction for Ultralong Cycling Li-Ion Battery Electrodes

Yongbiao Liu[1], Yang Song[2], Quanbing Lu[3], Linsen Zhang[3,4,*], Lulu Du[3], Shiying Yu[3], Yongshang Zhang[3,*]

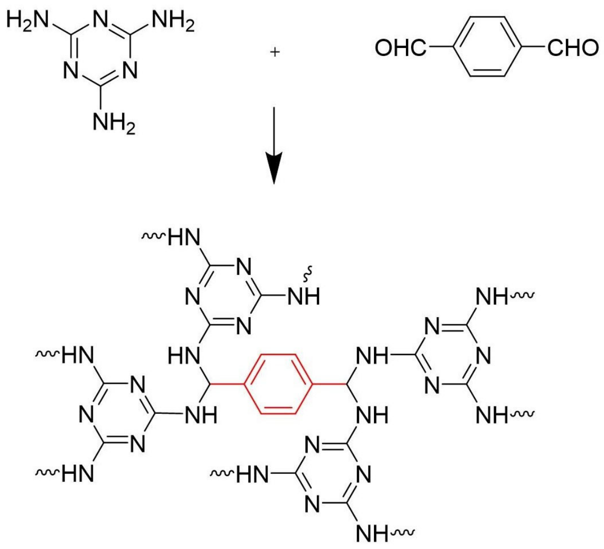

Figure S1. The synthesis route and chemical structure of COF.

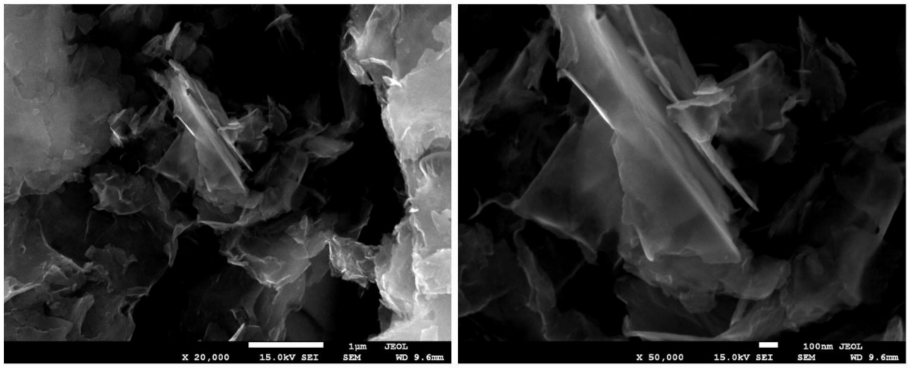

Figure S2. SEM images of Ti_3C_2−NH_2.

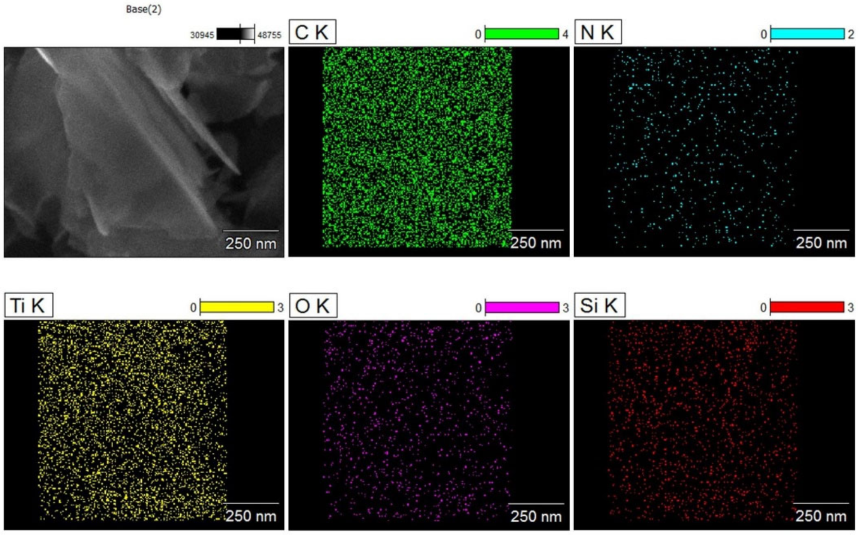

Figure S3. Elemental mapping of Ti_3C_2−NH_2.

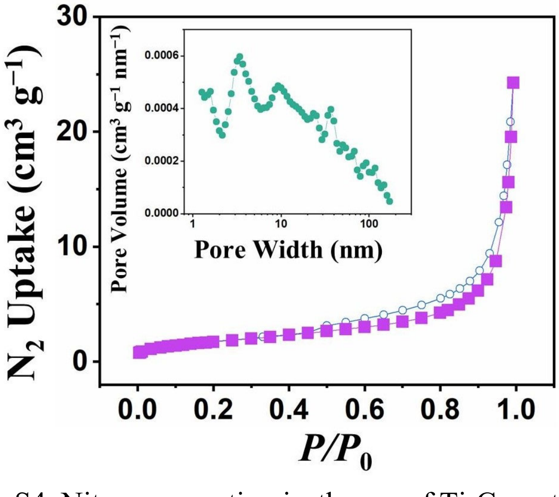

Figure S4. Nitrogen sorption isotherms of Ti_3C_2 materials.

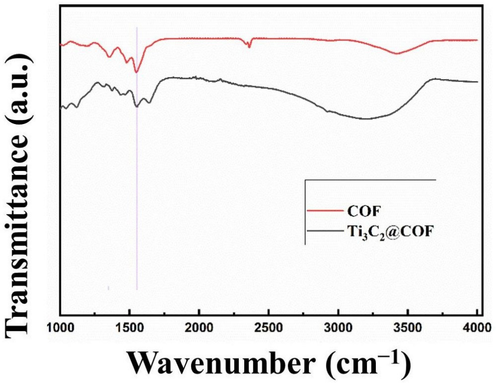

Figure S5. FTIR spectra of COF and Ti_3C_2@COF composite.

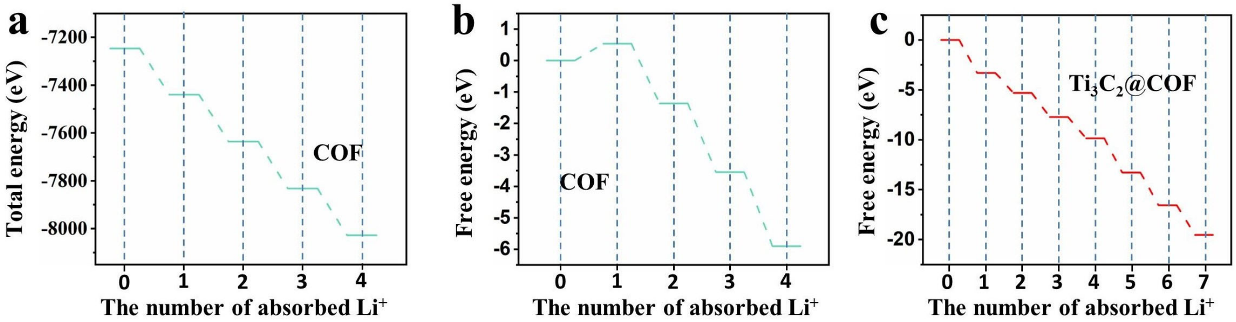

Figure S6. (a) Proposed lithiation pathway for the COF electrode. (b) The Gibbs free energies for lithiated models of COF + xLi (x=1-4). (c) The Gibbs free energies for lithiated models of Ti_3C_2@COF + xLi (x=1-7).

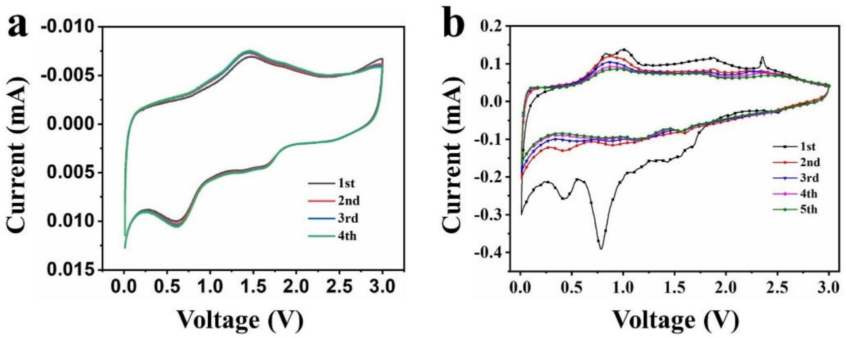

Figure S7. CV curves of (a) COF and (b) Ti_3C_2 electrode.

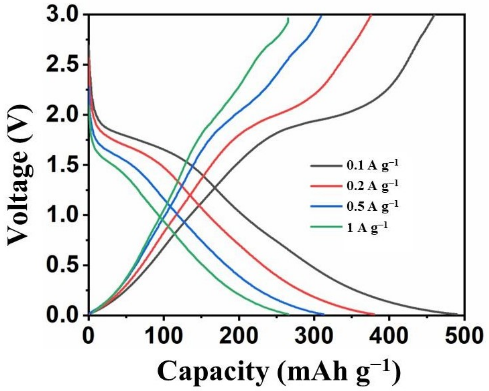

Figure S8. Galvanostatic charge-discharge curves of Ti_3C_2@COF electrode at different current density.
